# Liver Injury and Fibrosis Induced by Dietary Challenge in the Ossabaw Miniature Swine

**DOI:** 10.1371/journal.pone.0124173

**Published:** 2015-05-15

**Authors:** Tiebing Liang, Mouhamad Alloosh, Lauren N. Bell, Allison Fullenkamp, Romil Saxena, William Van Alstine, Phelan Bybee, Klára Werling, Michael Sturek, Naga Chalasani, Howard C. Masuoka

**Affiliations:** 1 Department of Medicine, Division of Gastroenterology/Hepatology, Indiana University School of Medicine, Indianapolis, Indiana, United States of America; 2 Department of Cellular & Integrative Physiology, Indiana University School of Medicine, Indianapolis, Indiana, United States of America; 3 Department of Pathology, Indiana University School of Medicine, Indianapolis, Indiana, United States of America; 4 School of Veterinary Medicine, Purdue University, Lafayette, Indiana, United States of America; 5 School of Biomedical Engineering, Purdue University, Lafayette, Indiana, United States of America; 6 2nd Department of Medicine, Semmelweis University, Budapest, Hungary; University of Catania, ITALY

## Abstract

**Background:**

Ossabaw miniature swine when fed a diet high in fructose, saturated fat and cholesterol (NASH diet) develop metabolic syndrome and nonalcoholic steatohepatitis (NASH) characterized by liver injury and fibrosis. This study was conducted to further characterize the development of NASH in this large animal model.

**Methods:**

Ossabaw swine were fed standard chow (control group; n = 6) or NASH diet (n = 6) for 24 weeks. Blood and liver tissue were collected and liver histology were characterized at 0, 8, 16 and 24 weeks of dietary intervention. Hepatic apoptosis and lipid levels were assessed at week 24.

**Results:**

The NASH diet group developed metabolic syndrome and progressive histologic features of NASH including: (a) hepatocyte ballooning at 8 weeks which progressed to extensive ballooning (>90% hepatocytes), (b) hepatic fibrosis at week 16, which progressed to moderate fibrosis, and (c) Kupffer cell accumulation with vacuolization at 8 weeks which progressed through week 24. The NASH diet group showed increased hepatocyte apoptosis that correlated with hepatic total and free cholesterol and free fatty acids, but not esterified cholesterol or triglycerides.

**Conclusions:**

This report further characterizes the progression of diet-induced NASH in the Ossabaw swine model. In Ossabaw swine fed the NASH diet: (a) hepatocyte injury and fibrosis can occur without macrovesicular steatosis or excess triglyceride accumulation; (b) hepatocyte ballooning generally precedes the development of fibrosis; (c) there is increased hepatocyte apoptosis, and it is correlated more significantly with hepatic free cholesterol than hepatic free fatty acids and had no correlation with hepatic triglycerides.

## Introduction

Nonalcoholic fatty liver disease (NAFLD) is the most common liver disease in the Western world, and it is characterized by intracellular lipid accumulation in hepatocytes that is not due to a secondary cause such as significant alcohol intake. The prevalence of NAFLD has been estimated at 20–34% in the general population, but it is considerably higher in certain populations such as individuals being evaluated for bariatric surgery [1,2].

NAFLD is composed of a range of disorders from relatively benign simple steatosis to nonalcoholic steatohepatitis (NASH), which is characterized by lobular inflammation, hepatocellular ballooning, and pericellular fibrosis that can progress to cirrhosis. NASH is predicted to become the predominant cause of cirrhosis requiring orthotopic liver transplantation in Western nations within the next two decades [3]. These features highlight the pressing need to improve our understanding of the pathophysiology and progression of NASH.

Animal models hold promise for improving our understanding of the pathogenesis of NASH and allow the testing of potential therapies. Rodent are widely employed, but there are some significant differences in the histopathologic and pathophysiologic features seen in rodent models and human NASH. Furthermore, it is difficult to conduct therapeutic investigations using novel compounds in these small animal models.

Ossabaw miniature swine, sharing many physiological similarities with humans, are an optimal and practical model for preclinical research into the pathophysiology of a variety of diseases including type 2 diabetes mellitus [4–6], metabolic syndrome [7,8], and NASH [9]. Ossabaw miniature swine are derived from feral swine that are descendants of swine from Spain released on Ossabaw Island off the coast of Georgia in the 16^th^ century. On this island, food is abundant in the fall with starvation conditions in the winter. The relative isolation of these swine resulted in a naturally selected “thrifty phenotype” that allows them to store large amounts of fat to survive the feast and famine ecology. This thrifty phenotype confers a propensity to long-term complications of food excess such as metabolic syndrome and related disorders [10]. Ossabaw miniature swine fed an excess calorie diet high in fat, cholesterol, and fructose develop insulin resistance [8,9,11,12]. Using dietary manipulation, we found that Ossabaw swine fed a “Western” or “NASH diet” develop severe metabolic syndrome with a markedly abnormal liver histology that mimics human NASH [9].

The current study focuses on further defining the disease progression in the Ossabaw swine fed the NASH diet as compared to a lean control group fed standard chow. At 0, 8, 16 and 24 weeks after initiating the dietary intervention, these animals were subjected to extensive phenotyping including liver histology. We quantified liver triglycerides, free fatty acids, and cholesterol and examined their relationship with hepatocyte apoptosis. Our primary aim was to develop a more dynamic picture of NASH development in these animals and make it useful for future clinical research.

## Materials and Methods

### Animal usage

All procedures performed for this study followed the guidelines of the Indiana University Animal Care and Use Committee and complied with the recommendations outlined by the National Research Council Principles of Laboratory Animal Care, and the American Veterinary Medical Association Panel on Euthanasia [13,14]. Twelve female Ossabaw miniature swine aged approximately six months at the start of the study were fed either standard chow (5L80) or NASH diet (5B4L) for 24 weeks.

### Experimental groups

Control group: Six swine received standard chow consisting of 18.5% calories from protein, 71% calories from complex carbohydrates, 10.5% calories from fat, and normal concentrations of methionine and choline (3500 ppm and 1500 ppm, respectively). These swine consumed an average of approximately 2300 kcal of standard chow per day. NASH diet group (n = 6) swine fed a fructose-based atherogenic diet (previously termed modified atherogenic diet [9] custom formulated by Purina TestDiet, Inc., Richmond, IN) providing 18% calories from fructose, 17% calories from protein (added casein), 43% calories from fat (admixture of partially hydrogenated soybean oil, coconut oil and lard), and methionine and choline at concentrations of 3500 ppm and 700 ppm, respectively [9]. These swine consumed an average of approximately 4500 kcal per day. Swine in both the control and NASH diet groups were given free access to 1000 grams of feed daily and unlimited access to water. Each of the swine completely consumed all of the provided feed within one hour.

### Physical measurements and sample collection

At baseline, week 8, week 16 and week 24 of dietary intervention, body weight and anatomical measurements were obtained, blood pressure was measured using a tail cuff sphygmomanometer, and an intravenous glucose tolerance test (IVGTT) was performed using a previously described method [11,12,15,16]. Briefly, swine were feed deprived for 8 hours and then subjected to blood draw and an IVGTT. Glucose (0.5 g/kg) was injected intravenously and arterial blood samples were obtained before (-5 min) and at 5, 10, 20, 30, 40, 50, and 60 min after the injection. Plasma was frozen in liquid nitrogen, then stored at -80°C until analysis for glucose (glucose oxidase; Sigma-Aldrich, St. Louis, MO) and insulin (Millipore, Billerica, MA). Serum samples were collected at weeks 0, 8, 16 and 24 and liver samples were obtained percutaneously at weeks 0, 8 and 16 weeks and surgically prior to sacrifice at week 24. At week 24, animals were sacrificed via excision of the heart under general anesthesia according to a previously described protocol [17]. Liver biochemistries, glycemic measurements, and lipid profiles were measured in fasting serum samples by Antech (Antech Diagnostics, Fishers, IN) at baseline and at weeks 8, 16 and 24.

### Tissue preparation and histological grading

The liver cores obtained at the biopsy time points (baseline, week 8 and week 16) and a portion of the left lobe of the liver obtained at sacrifice (week 24) were flash-frozen and stored at -80°C or fixed in formalin, processed and embedded in paraffin for subsequent hematoxylin and eosin (H&E), trichrome, oil red-O and Periodic acid-Schiff staining. The slides were examined by light microscopy and blindly scored by one human and one veterinary hepatopathologist as described previously [9].

### Hepatic fatty acids, cholesterol, triglycerides, and apoptotic measurements

Liver samples obtained at week 24 were employed. 100 mg of liver tissue was placed in 1 ml of PBS and homogenized using a Polytron Model PT 10–35 GT-D homogenizer (Kinematica, Luzern, Switzerland). The homogenate was then centrifuged at 10,000 x g for 5 minutes at 4 C and the supernatant retained for the assays. Liver triglycerides, free fatty acids, total and free cholesterol were measured using colorimetric assay kits per the manufacturer’s protocol (Biovision, Milpitas, CA). Caspase 3/7 activity was measured using the Apo-ONE Homogeneous Caspase-3/7 assay per the manufacture’s protocol (Promega, Madison, WI). Terminal deoxynucleotidyl transferase dUTP nick end labeling (TUNEL) assay was performed on a paraffin embedded sections using the fluorescein In Situ Cell Death Detection Kit, POD combined with DAB substrate per the manufacturer’s protocol (Roche Applied Science, Indianapolis, IN). Hepatocyte apoptosis was quantified by counting the number of TUNEL positive cells in twenty representative 200x microscopic fields across the section and the total number of cells per field was determined by counting the number of nuclei present in a representative quadrant in each field and multiplying by four. The apoptotic index was calculated as the number of TUNEL positive cells per 1000 cells.

### Data analysis

Data are expressed as mean ± standard deviation. Student’s t-test was used to detect significant differences between two intervention groups. Significant differences between different time points and baseline were also compared within each group. Linear mixed effect with random intercept and slope model was used for intravenous glucose tolerance test (IVGTT) data analysis. Data from -5 min and 0 min baseline time points were averaged and subtracted from other time points. In an attempt to avoid a type II interpretive error (a false negative), significance was reported at the *P* <0.05 and *P* <0.10 levels [18].

## Results

### Body characteristics

Phenotypic characteristics of the swine are displayed in Table 1. At the 8, 16, and 24 week time points, the NASH diet group weighed significantly more than the control group (Table 1. p = 0.04, p = 0.02, p = 0.026, respectively). Mean body circumference (a measurement analogous to waist circumference) was significantly increased in the NASH diet group compared to the control group at weeks 8 and 16 (p = 0.002 and 0.012, respectively) (S1 Table). Compared to the control group, the NASH diet group had significantly increased systolic (p = 0.01 and p = 0.003, respectively) and diastolic blood pressure at weeks 16 and 24 (p<0.001 and p = 0.014, respectively). Liver weight at week 24 was significantly higher in swine fed NASH diet compared to controls (2073 ± 921 vs. 844 ± 191 gm, p = 0.01).

**Table 1:** Selected characteristics of swine in the control group (n = 6) and NASH diet group (n = 6) at baseline and at weeks 8, 16 and 24 of dietary intervention.

	Baseline			Week 8				Week 16				Week 24			
	control	NASH	^§^P	control	NASH	^§^P	*p	control	NASH	^§^P	*p	control	NASH	^§^P	*p
**Weight (Kg)**	45.3 ± 5.8	46.8 ± 5.3	0.7	52.4 ± 5.7	64.1 ± 7.2	**0.04**	**0.004**	58.1 ± 5.2	82.5 ± 15.6	**0.020**	**0.002**	64.6 ± 5.7	93.2 ± 20.4	**0.026**	**0.002**
**Blood pressure (mmHg)**															
**Systolic**	92 ± 33	103 ± 25	0.5	118 ± 24	127 ± 15.5	0.4	0.07	114 ± 8	135 ± 15	**0.012**	**0.024**	116 ± 12	147 ± 15	**0.003**	**0.005**
**Diastolic**	60 ± 26	69 ± 15	0.5	79 ± 21	83 ± 14	0.6	0.12	72 ± 5	97 ± 13	**0.001**	**0.005**	75 ±11	99 ± 16	**0.014**	**0.008**
**Serum biochemistry**															
**AST (IU/L)**	32±5	25±5	0.3	37±12	85±20	0.1	**0.03**	32±3.5	94±26	0.06	**0.03**	31±5	69±10	**0.04**	**0.001**
**ALT (IU/L)**	29±2	27±2	0.6	37±10	77±19	0.2	**0.04**	27±2	72±20	0.09	0.06	28±3	64±13	0.06	**0.030**
**Cholesterol (mg/dL)**	83±10	64±3	0.1	149±74	571±116	0.07	**0.007**	69±7	747±223	0.028	**0.03**	79±7	610±121	0.006	**0.006**
**Triglyceride (mg/dL)**	34±5.4	32±8	0.8	36±5	61±30	0.4	0.3	48.5±25	64±16	0.7	**0.03**	35±4	92±29	0.08	0.08
**Insulin (µU/mL)**	16.2±3.3	8.9±1.2	0.06	8.6±1.1	8.7±1.4	1.0	0.9	13.5±3.8	8.1±1.1	0.2	0.7	11.1±1.5	10.7±1.2	0.9	0.4
**HOMA-IR**	4.4±1.6	1.7±0.2	0.1	1.8±0.3	1.5±0.3	0.5	0.7	2.7±0.9	1.5±0.2	0.236	0.7	3.0±0.5	2.8±0.2	0.8	**0.05**

Phenotypic characteristics of the swine. Weight, blood pressure, and serum biochemistries at baseline and at the 8, 16, and 24 week time points. The NASH diet group weighed significantly more than the control group. Compared to the control group, the NASH diet group had significantly increased systolic and diastolic blood pressure at weeks 16 and 24. The NASH diet group displayed transaminase elevations after 16 weeks of feeding and a trend of increasing serum AST and ALT levels (p<0.1) was also observed. At week 24, serum AST, ALT, triglyceride levels in the NASH diet group were more than double those of control swine. While serum cholesterol progressively increased in the NASH diet group. At week 24, serum cholesterol in the NASH diet group was dramatically higher than in the control group.

§P is the p-value between NASH and control groups at the indicated time points.

*P is the P-value of within NASH group comparison between the indicated time points and baseline.

### Serum chemistry

The NASH diet group displayed transaminase elevations after 16 weeks of feeding and a trend of increasing serum AST and ALT levels (p<0.1) was also observed (Table 1). At week 24, serum AST, ALT, triglyceride levels in the NASH diet group were more than double those of control swine (Table 1). While serum cholesterol progressively increased in the NASH diet group. At week 24, serum cholesterol in the NASH diet group was dramatically higher than in the control group (610.5 ± 121 vs. 79 ± 7 mg/dL, p = 0.006) (Table 1).

### Serum glycemic measures by IVGTT

**Fig 1:**
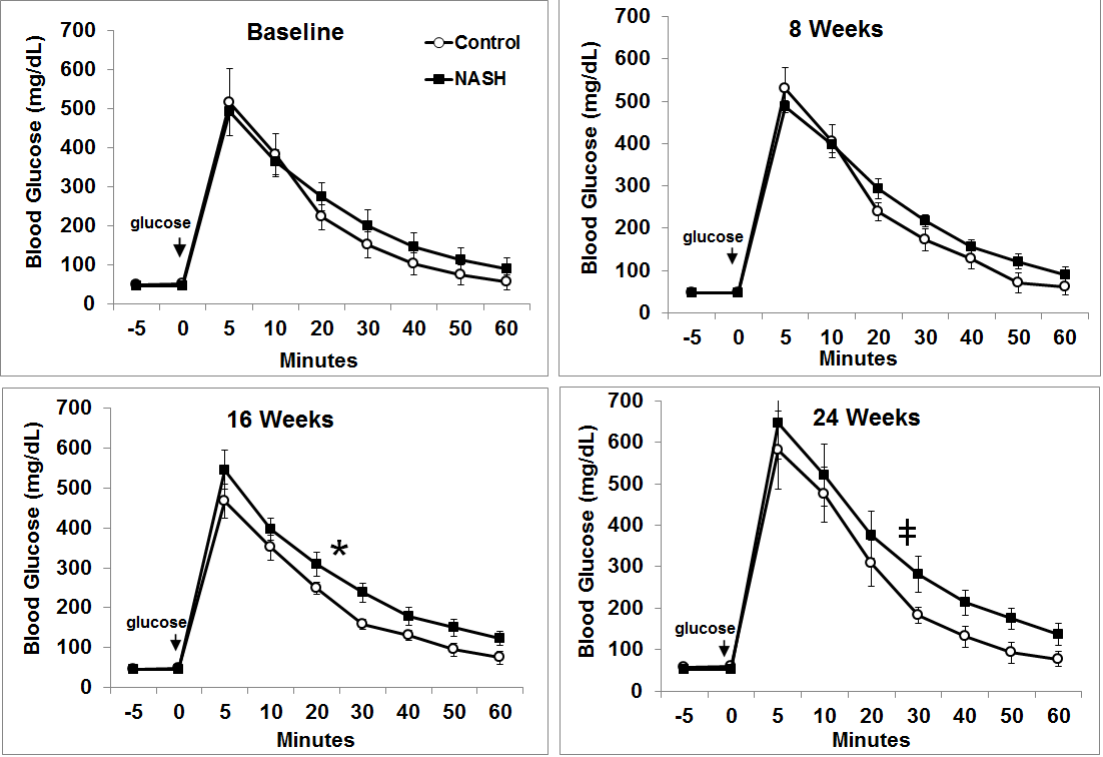
IV glucose tolerance tests of control and NASH diet swine. Time is indicated as minutes following glucose injection. Blood glucose during the first 60 minutes after IV glucose challenge was higher in swine fed NASH diet at weeks 16 and 24.

Blood glucose levels for 60 minutes following IV glucose challenge were not different at baseline (p = 0.7) and at week 8 (p = 0.9) between two groups. Blood glucose levels were higher in the NASH diet group with statistical significance at week 16 (p = 0.009) and borderline significance at week 24 (p = 0.1) (Fig 1). While serum insulin levels did not change significantly in either group throughout the intervention period, swine in the NASH diet group had significantly higher HOMA-IR score at week 24 compared to baseline (Table 1).

### Liver histology

**Fig 2:**
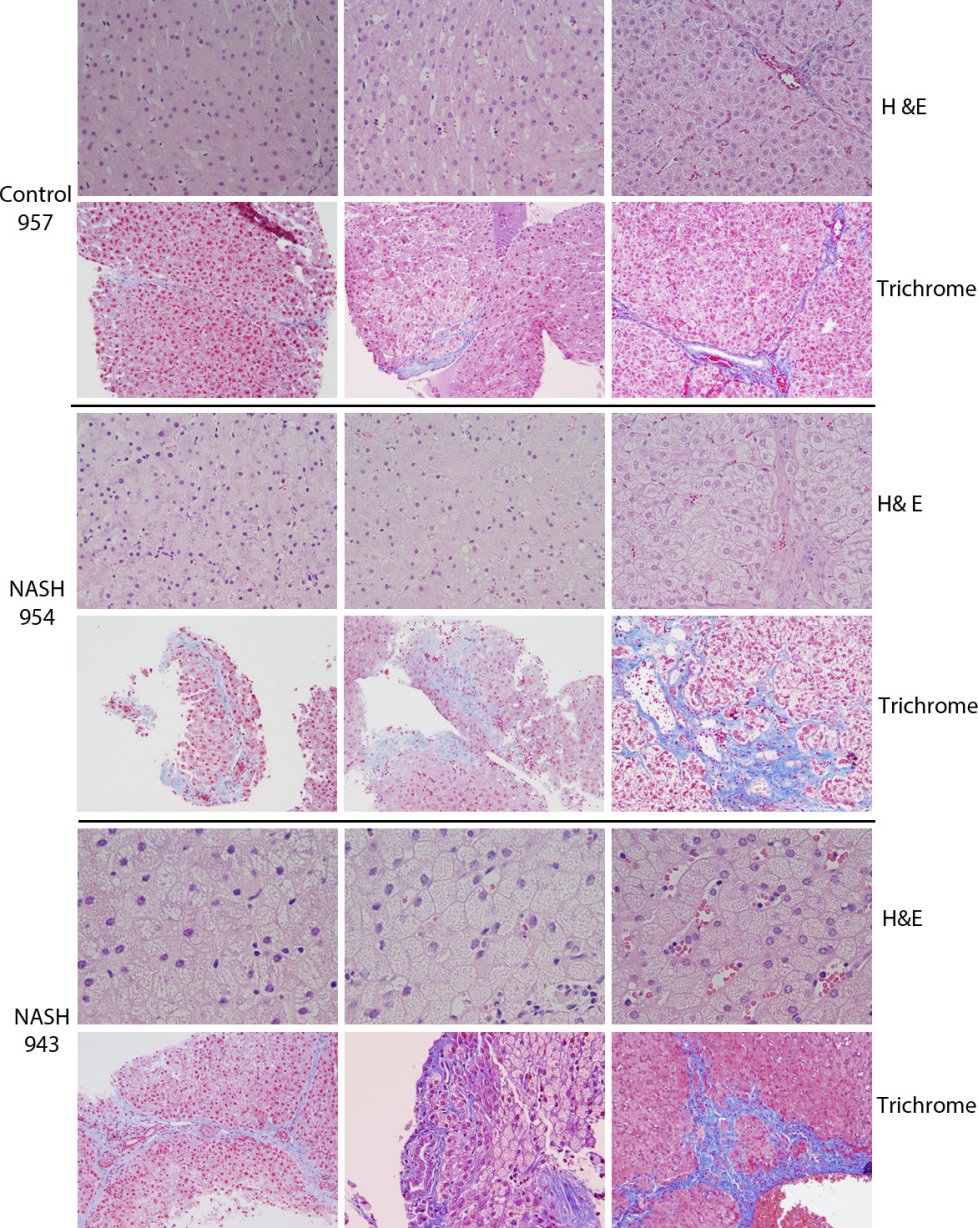
Liver histology of representative control diet (control swine # 957) and NASH diet swine (NASH diet # 954 and 943). For each animal, top row is H&E and bottom row is trichrome staining; the columns are weeks 8, 16 and 24, respectively. All sections are at 200x magnification except for all week 24 H&E staining, and week 8 and 16 for NASH 943 which are 400x magnification. Control 957 swine showed no evidence of liver injury or fibrosis throughout the experiment. Livers from swine fed NASH diet showed progressive hepatocyte ballooning degeneration (as evidenced by wispy clear cytoplasm), Kupffer cell accumulation, and fibrosis (indicated by an arrow).

**Fig 3:**
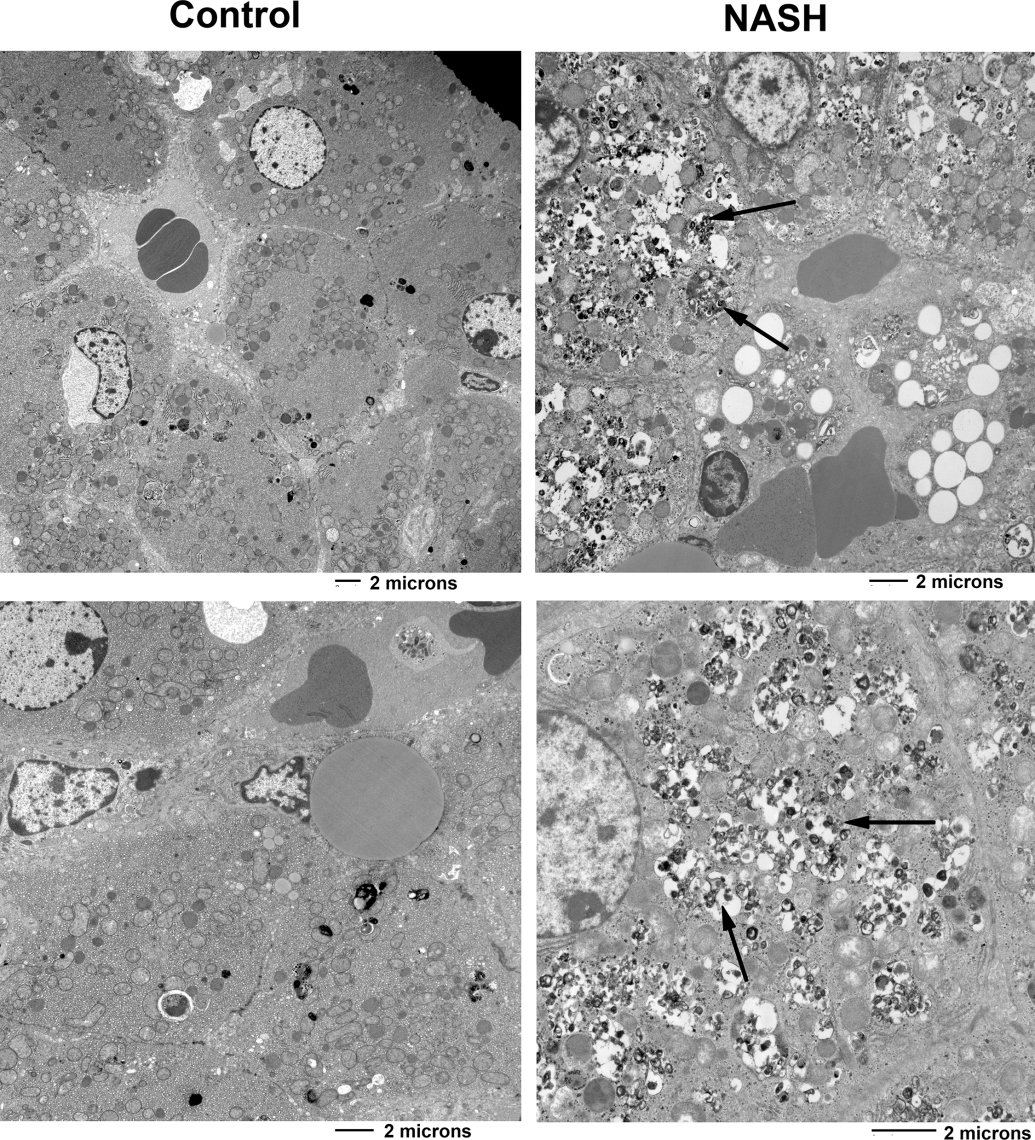
Liver histology by electron microscopy (EM) (A-D 21,400X). **A and C:** An EM section from a control swine hepatocyte at week 24 shows no accumulation of abnormal material. **B and D**: An EM section of the liver from a swine fed NASH diet at week 24. Hepatocytes were filled with electron-dense material arranged as whorled lamellar structures, located within membrane-bound vesicles (white arrows) consistent with autophagolysosomes.

Serial liver biopsies of swine in the NASH diet group demonstrated progressive histological changes as described in Table 2 and Fig 2. Feathery and enlarged hepatocytes, consistent with hepatocyte ballooning, started to appear at week 8 and became more prominent at weeks 16 and 24. By week 24, five out of six swine receiving NASH diet exhibiting extensive hepatocyte ballooning (>90% of the hepatocytes). Kupffer cell vacuolization was first observed at week 8, and it became progressively more prominent at weeks 16 and 24. Pericellular fibrosis was first evident at week 16 and it became more prominent at week 24. At week 16, 3 out of 6 swine in the NASH diet group exhibited mild fibrosis but by week 24, 5 out of 6 swine showed moderate fibrosis (Fig 2). Of note, the NASH diet group demonstrated no evidence of significant macrovesicular steatosis or lobular inflammation throughout the dietary intervention. Liver histology of swine in the control group remained largely unchanged throughout the intervention period. Electron microscopic examination at week 24 revealed normal hepatocytes in control group (Fig 3A and 3C), but swine hepatocytes in the NASH diet group had copious membrane-bound vesicles consistent with autophagolysosomes filled with electron-dense material arranged as whorled lamellar structures (Fig 3B and 3D).

**Table 2:** Histologic features of NASH in serial liver biopsies of both groups of swine.

	Baseline	Week 8	Week 16	Week 24
**CONTROL**				
Macrovesicular steatosis	0/4	0/6	0/6	0/6
Hepatocyte ballooning	0/4	1*/6	0/6	1*/6
Kupffer cell vacuolization	0/5	0/6	0/6	0/6
Lobular Inflammation	1/5	0/6	0/6	0/6
Extramedullary hematopoiesis	0/4	0/6	0/6	1/6
Fibrosis	0/4	0/6	0/6	0/6
**NASH**				
Macrovesicular steatosis	0/3	1/6	0/6	0/6
Hepatocyte ballooning	1/5	1/6	4/6	5/6
Kupffer Cell vacuolization	1/5	5/6	5/6	5/6
Lobular Inflammation	0/3	0/6	0/6	0/6
Extramedullary hematopoiesis	1/3	0/6	1/6	5/6
Fibrosis	1/3	1/6	3/6	5/6

¶Several percutaneous liver biopsy specimens from baseline were gelatinous and bloody precluding adequate examination

*One swine in the control group at week 8 and another swine in the control group at week 24 showed <5% hepatocyte ballooning. The NASH diet group demonstrated progressive histological changes of NASH. In the NASH group, feathery and enlarged hepatocytes, consistent with hepatocyte ballooning, started to appear at week 8 and became more prominent at weeks 16 and 24. By week 24, five out of six swine receiving NASH diet exhibiting extensive hepatocyte ballooning (>90% of the hepatocytes). Kupffer cell vacuolization was first observed at week 8, and it became progressively more prominent at weeks 16 and 24. Pericellular fibrosis was first evident at week 16 and it became more prominent at week 24. At week 16, 3 out of 6 swine in the NASH diet group exhibited mild fibrosis and by week 24, 5 out of 6 swine showed moderate fibrosis. Of note, the NASH diet group demonstrated no evidence of significant macrovesicular steatosis or lobular inflammation throughout the dietary intervention. Liver histology of swine in the control group remained unchanged throughout the intervention period.

### Apoptosis

**Fig 4:**
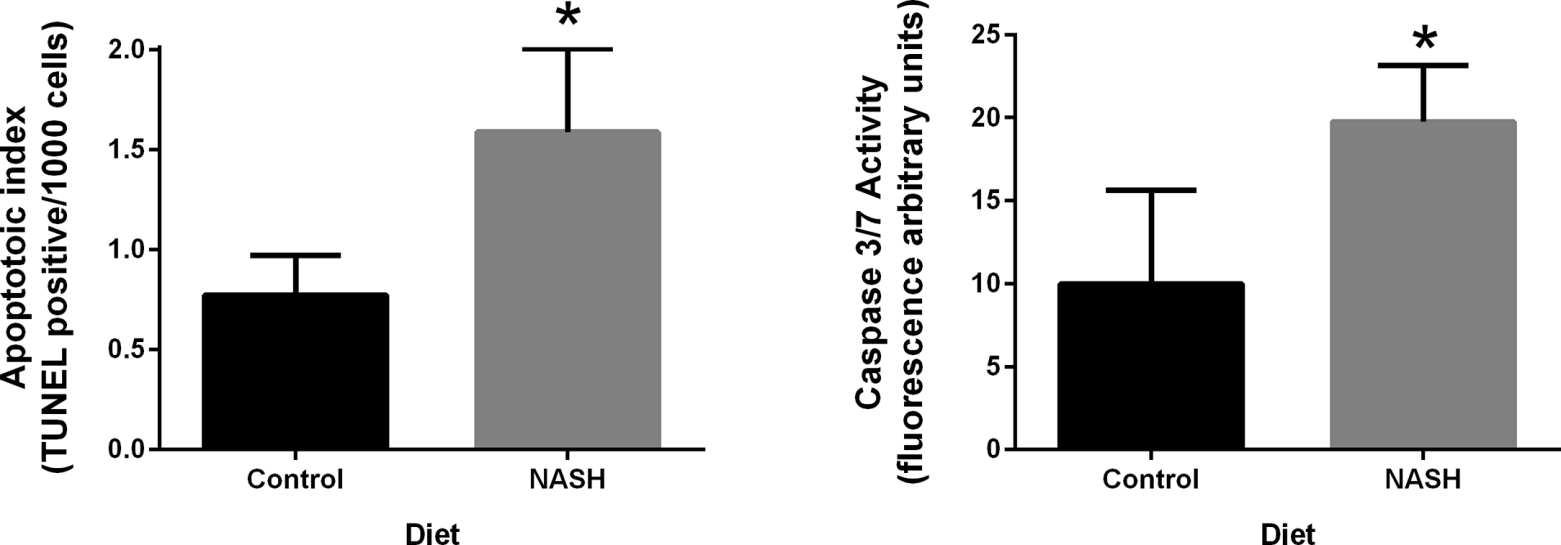
Quantification of hepatic apoptosis at week 24. **A:** TUNEL assay was performed on paraffin embedded sections. The NASH diet group had significantly higher apoptotic index (number of TUNEL positive cells per 1000 cells) (p = 0.0125). **B:** Hepatic caspase 3/7 activity shown in fluorescence arbitrary units. Caspase 3/7 activity is significantly higher in NASH diet group than the control diet fed group (p = 0.0397). All data was presented as Mean ± SEM. * indicates statistically significance difference between the NASH diet and the control diet groups.

Apoptosis was measured in the liver at week 24 through TUNEL assay which detects nuclear DNA fragmentation which is an important hallmark of the late stages of apoptosis. The hepatic apoptotic index (TUNEL positive cells per 1000 cells) was significantly higher in the NASH diet group compared to swine in the control group (Fig 4A) (p = 0.0125). Similarly, hepatic caspase 3/7 activity at week 24 was significantly higher in the NASH diet group compared to the control group (Fig 4B) (p = 0.0397).

### Liver lipid measurements

Changes in hepatocyte lipid species have been proposed to play an important pathophysiologic role in NASH. To further examine this we determined the level of total cholesterol, free cholesterol, esterified cholesterol, free fatty acids, and triglycerides in the liver at week 24 (Table 3). These measurements could not be performed at other points because insufficient liver tissue was available from percutaneous liver biopsy.

**Table 3:** Liver Lipid Quantification in NASH and Control Swine at Week 24.

	NASH diet group (n = 6)	Control group (n = 6)	p value
Free fatty acids (pmol/mg liver)	144.7 ±137	28.1 ± 22	0.09
Triglycerides (nmol/mg liver)	76.3 ± 28	50.5 ± 39	0.2
Total Cholesterol (μg/mg liver)	10.4 ± 1.5	2.6 ± 0.2	<0.0001
Free Cholesterol (μg/mg liver)	6.2 ± 3.4	2.05 ± 0.4	0.03
Esterified Cholesterol (μg/mg liver)	4.22 ± 4.04	0.59 ± 0.33	0.08

Quantification of lipid species in livers of NASH and control group swine at Week 24 of dietary intervention. Shown is the mean ± standard deviation.

**Fig 5:**
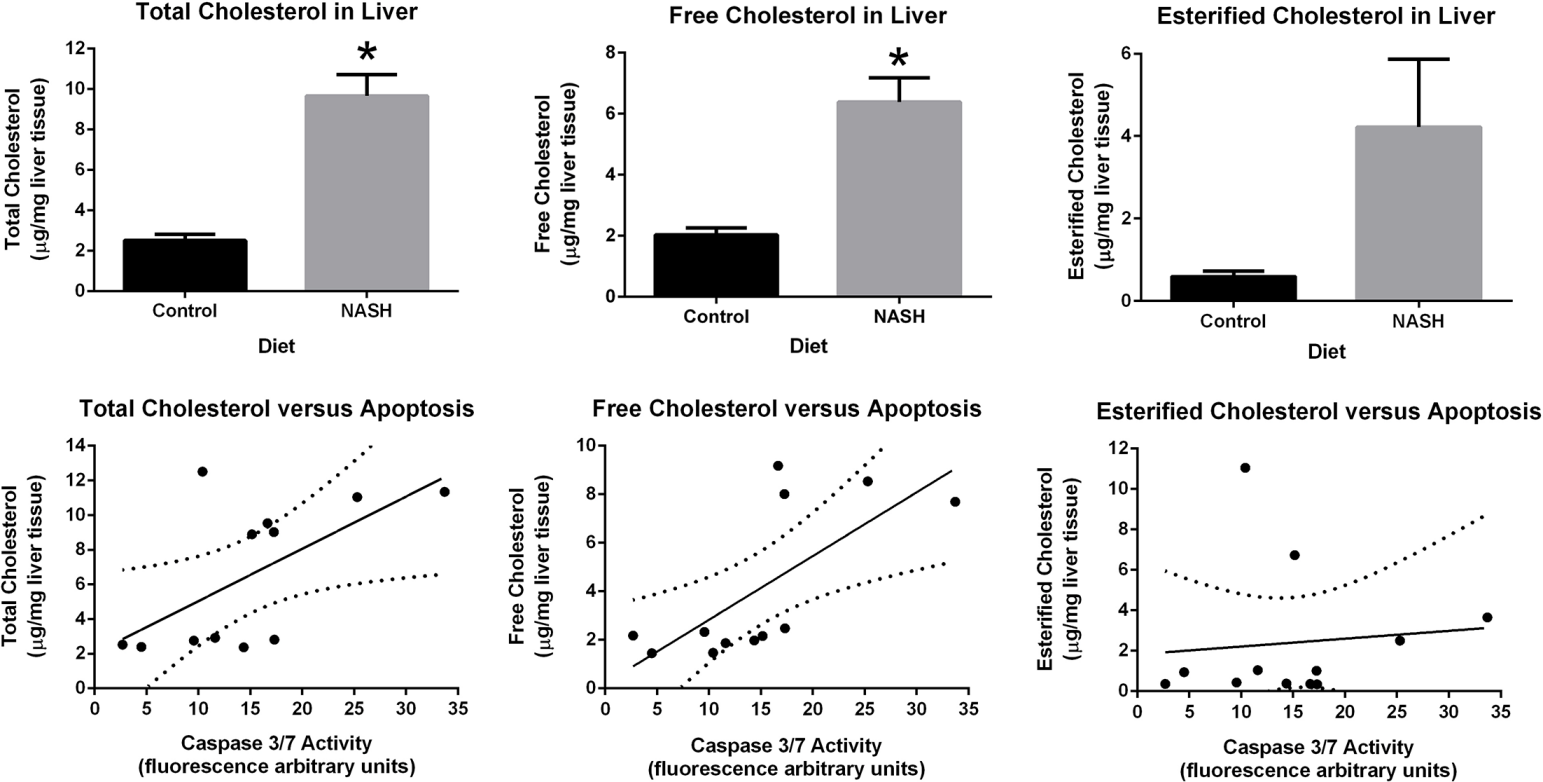
Liver total, free, and esterified cholesterol measurement and their correlation with liver caspase 3/7 activity at week 24. Total cholesterol **(A)**, free cholesterol **(B)** and esterified cholesterol **(C)** were higher in the NASH diet fed group than control group. Positive correlations were observed between caspase 3/7 activity and total cholesterol (R^2^ = 0.3770, p = 0.0338) **(D)** and free cholesterol (R^2^ = 0.4961, p = 0.0105) **(E)** levels, but not esterified cholesterol (R^2^ = 0.0098, p = 0.7593) **(F)**. * indicates statistically significance difference between the NASH diet and the control diet groups.

**Fig 6:**
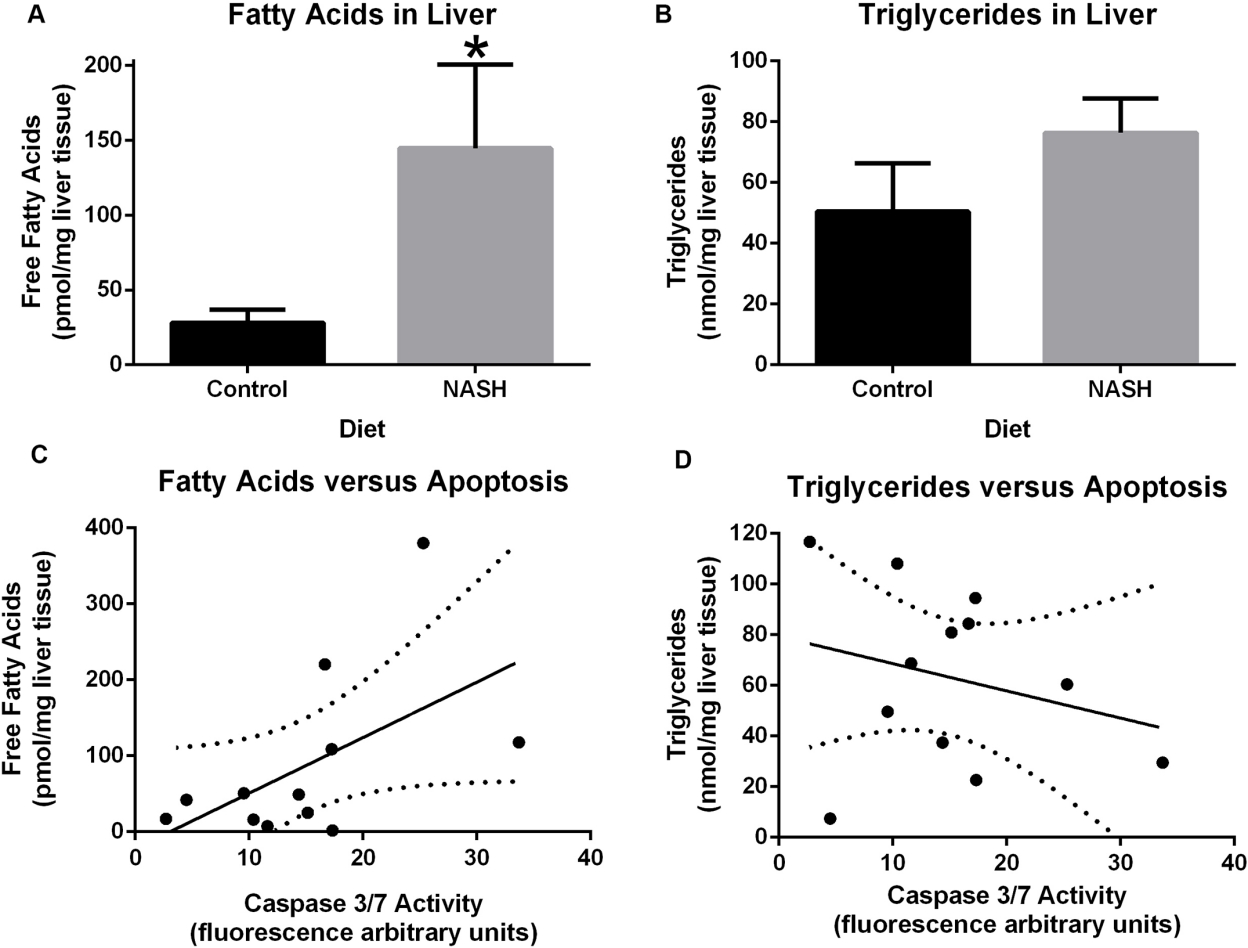
Correlation of hepatic free Fatty acid and triglyceride levels with apoptosis at week 24. **A:** Hepatic free fatty acid levels were more than 5-fold higher in the NASH diet group than control group although it did not reach statistical significance (p = 0.0667). **B:** There was no difference in hepatic triglyceride concentration between two groups (p = 0.2134). **C**: A positive correlation was observed between caspase 3/7 activity and fatty acid levels (R^2^ = 0.3096, p = 0.0603). **D:** There was no correlation between hepatic triglyceride concentration and hepatic caspase 3/7 activity (R^2^ = 0.0684, p = 0.4115).

Liver cholesterol levels were nearly four-fold higher in the NASH diet group compared to control group (10.4 ± 1.5 vs 2.6 ± 0.23 nmol/mg, p<0.0001) (Fig 5A). Liver free cholesterol was significantly higher in the NASH diet group (6.2 ± 3.4 vs 2.0 ± 0.4 μg/mg, p = 0.03) (Fig 5B) and esterified cholesterol was also higher in the NASH diet group but this difference was of borderline statistical significance (4.22 ± 4.04 versus 0.59 ± 0.33 μg/mg, p = 0.08) (Fig 5C). Hepatic free fatty acid levels were higher in the NASH diet group but this difference did not reach statistical significance (144.7 ± 137 versus 28 ± 22, p = 0.09) (Fig 6A). As described previously [17], hepatic triglyceride concentration was not significantly different between the two groups (76.3 ± 28 versus 50.5 ± 38. 8, p = 0.2) (Fig 6B).

Hepatic caspase 3/7 activity significantly correlated with total (R^2^ = 0.37, p = 0.03) and free hepatic cholesterol (R^2^ = 0.49, p = 0.01) but not with esterified cholesterol (R^2^ = 0.01, p = 0.76) (Fig 5D, 5E, and 5F). Hepatic caspase 3/7 activity had no correlation with hepatic triglyceride levels (R^2^ = 0.07, p = 0.41) but a trend towards correlation with hepatic free fatty acids (R^2^ = 0.31, p = 0.06) (Fig 6C and 6D).

## Discussion

This study confirms that Ossabaw swine fed NASH diet consisting of excessive fructose, saturated fat and cholesterol is a reproducible model for inducing liver cell injury and fibrosis associated with metabolic syndrome (obesity, dyslipidemia, hypertension, and glucose intolerance) in a large animal. Although not described in this paper, these animals developed severe truncal obesity and extensive coronary artery disease, two additional features of NAFLD in humans. There are numerous small animal models of NAFLD, NASH and even hepatocellular carcinoma described in the literature [19], but a large animal model, while expensive and difficult to develop, offers certain benefits such as the ability to perform serial liver biopsies, investigating multiple organ systems simultaneously, and test novel agents to prevent or reverse NASH [20,21]. In addition, a large animal model is essential to obtain Food and Drug Administration approval for clinical studies in humans [22].

The main observations in this study are that: (a) hepatocyte injury and fibrosis in response to NASH diet can occur in the absence of macrovesicular steatosis or excess triglyceride accumulation; (b) in general, hepatocyte ballooning precedes the development of fibrosis; (c) there is increased hepatocyte apoptosis in the animals fed the NASH diet; and (d) hepatocyte apoptosis correlated more significantly with hepatic free cholesterol than hepatic free fatty acids and had no correlation with hepatic triglycerides.

There is an evolving body of literature suggesting that hepatic triglyceride accumulation is not relevant in the overall pathogenesis of steatohepatitis and progressive fibrosis. Our study provides compelling evidence supporting this notion. To our knowledge, our animal model may be the first of its kind to exhibit extensive cell injury and fibrosis and yet not show significant hepatic macrovesicular steatosis. It remains to be determined whether longer duration studies in the Ossabaw swine model would reveal macrovesicular steatosis which is one of its defining components human NAFLD syndrome.

Although it is long believed that fibrosis is a long term consequence of hepatocyte injury, it is possible that lipotoxic mediators such as free fatty acids and free cholesterol may injure hepatocytes and may simultaneously activate stellate cells. Therefore we explored the temporal relationship of fibrosis with hepatocyte ballooning and found that in general ballooning preceded fibrosis and severity of fibrosis correlated with severity of ballooning.

Apoptosis was significantly increased in the livers of swine fed the NASH diet compared with controls based on the TUNEL assay and caspase 3/7 measurement reinforcing the hypothesis that apoptosis plays an important pathophysiologic role in NASH. Increased hepatocyte apoptosis was correlated with increased liver free fatty acids, total cholesterol, and free cholesterol, but not with esterified cholesterol or liver triglyceride levels. The results highlight the important role of free fatty acids and free cholesterol in mediating hepatocyte injury in NASH and lend support to the concept that triglycerides and esterified cholesterol are not the mediators of hepatocyte injury.

Two additional features of our swine model are extensive Kupffer cell accumulation in the liver tissue of swine fed NASH diet and electron dense material in the hepatocytes by electron microscopy (EM). While Kupffer cells may not be readily evident on routine H&E staining of liver samples from patients with NASH, their accumulation is a common feature among rodent models of steatosis induced by cholesterol atherogenic diets. In our swine fed NASH diet, we observed vacuolated Kupffer cells accumulate as early as 8 weeks and their accumulation progressed through 24 weeks. It is unclear if these vacuoles represent cholesterol and cholesterol crystal accumulation in the Kupffer cells as it previously has been described in some rodent models of NASH. Despite this extensive Kupffer cell accumulation, it was surprising that little hepatic inflammation was observed in these swine. Electron dense material in the hepatocytes appears to be autophagolysosomes, and although we did not specifically characterize it we speculate this material represents cholesterol and cholesterol esters.

A limitation of this study was the small sample size of each group (N = 6). Although the swine model is the preferred model to use for this study because serial biopsies can be obtained from the same animal, there are budgetary restraints that limit the number of large animals that can be studied. Because there was large variation in some measurements, we reported significance at *P* <0.05 and a trend of difference at *P* <0.10 levels to avoid a type II interpretive error (a false negative) [18].

In summary, this report further characterizes a more detailed time course of liver injury and fibrosis induced by NASH diet in the Ossabaw swine model. These data provide a solid, fundamental basis for future studies to better understand the mechanisms of cell injury and fibrosis in this model.

## Supporting Information

S1 Table Morphometric characteristics of swine in the control group (n = 6) and NASH diet group (n = 6) at baseline and at weeks 8, 16 and 24. Morphometric characteristics of swine in the control group (n = 6) and NASH diet group (n = 6) at baseline and at weeks 8, 16 and 24. Mean body circumference (a measurement analogous to waist circumference) was significantly increased in the NASH diet group compared to the control group at weeks 8 and 16 (p = 0.002 and 0.012, respectively). Shown is the mean ± standard deviation.
(DOCX)

## References

[1] Browning JD, Szczepaniak LS, Dobbins R, Nuremberg P, Horton JD, Cohen JC, et al (2004) Prevalence of hepatic steatosis in an urban population in the United States: impact of ethnicity. Hepatology 40: 1387–1395. 15565570 10.1002/hep.20466

[2] Beymer C, Kowdley KV, Larson A, Edmonson P, Dellinger EP, Flum DR (2003) Prevalence and predictors of asymptomatic liver disease in patients undergoing gastric bypass surgery. Arch Surg 138: 1240–1244. 14609874 10.1001/archsurg.138.11.1240

[3] Charlton MR, Burns JM, Pedersen RA, Watt KD, Heimbach JK, Dierkhising RA (2011) Frequency and outcomes of liver transplantation for nonalcoholic steatohepatitis in the United States. Gastroenterology 141: 1249–1253. 10.1053/j.gastro.2011.06.061 21726509

[4] Buhlinger CA, Wangsness PJ, Martin RJ, Ziegler JH (1978) Body composition, in vitro lipid metabolism and skeletal muscle characteristics in fast-growing, lean and in slow-growing, obese pigs at equal age and weight. Growth 42: 225–236. 680583

[5] Cote PJ, Wangsness PJ, Varela-Alvarez H, Griel LC Jr., Kavanaugh JF (1982) Glucose turnover in fast-growing, lean and in slow-growing, obese swine. J Anim Sci 54: 89–94. 7085505 10.2527/jas1982.54189x

[6] Wangsness PJ, Martin RJ, Gatchel BB (1980) Insulin induced growth hormone response in fast-growing, lean and in slow-growing, obese pigs. Growth 44: 318–326. 7014390

[7] Trask AJ, Katz PS, Kelly AP, Galantowicz ML, Cismowski MJ, West TA, et al (2012) Dynamic micro- and macrovasscular remodeling in coronary circulation of obese Ossabaw pigs with metabolic syndrome. J Appl Physiol (1985) 113: 128–1140. 10.1152/japplphysiol.00604.2012 PMC3487495 22837170

[8] Dyson MC, Alloosh M, Vuchetich JP, Mokelke EA, Sturek M (2006) Components of metabolic syndrome and coronary artery disease in female Ossabaw swine fed excess atherogenic diet. Comp Med 56: 35–45. 16521858

[9] Lee L, Alloosh M, Saxena R, Van Alstine W, Watkins BA, Klaunig JE, et al (2009) Nutritional model of steatohepatitis and metabolic syndrome in the Ossabaw miniature swine. Hepatology 50: 56–67. 10.1002/hep.22904 19434740 PMC3254146

[10] Neel JV (1962) Diabetes mellitus: a "thrifty" genotype rendered detrimental by "progress"? Am J Hum Genet 14: 353–362. 13937884 PMC1932342

[11] Edwards JM, Neeb ZP, Alloosh MA, Long X, Bratz IN, Peller CR, et al (2010) Exercise training decreases store-operated Ca2+entry associated with metabolic syndrome and coronary atherosclerosis. Cardiovasc Res 85: 631–640. 10.1093/cvr/cvp308 19744946 PMC2802199

[12] Sturek M, Alloosh M, Wenzel JW, Byrd JP, Edwards JM, Lloyd PG, et al (2007) Ossabaw Island miniature swine: cardiometabolic syndrome assessment In: Swindle MM, editor. Swine in the Laboratory: Surgery, Anesthesia, Imaging, and Experimental Techniques. 2nd ed. Boca Raton, FL: CRC Press pp. 397–400.

[13] (2011) Guide for the Care and Use of Laboratory Animals: Eighth Edition: The National Academies Press. 21595115

[14] Association APoEAVM (2001) 2000 Report of the AVMA Panel on Euthanasia. J Am Vet Med Assoc 218: 669–696. 11280396 10.2460/javma.2001.218.669

[15] Neeb ZP, Edwards JM, Alloosh M, Long X, Mokelke EA, Sturek M (2010) Metabolic syndrome and coronary artery disease in Ossabaw compared with Yucatan swine. Comp Med 60: 300–315. 20819380 PMC2930329

[16] Stoll B, Horst DA, Cui L, Chang X, Ellis KJ, Hadsell DL, et al (2010) Chronic parenteral nutrition induces hepatic inflammation, steatosis, and insulin resistance in neonatal pigs. J Nutr 140: 2193–2200. 10.3945/jn.110.125799 20980637 PMC2981005

[17] Nolen RS (2011) AVMA board approves Panel on Euthanasia report: updated guidelines cover more species and methods. J Am Vet Med Assoc 239: 1269 22164532

[18] Williams JL, Hathaway CA, Kloster KL, Layne BH (1997) Low power, type II errors, and other statistical problems in recent cardiovascular research. Am J Physiol 273: H487–493. 9249522 10.1152/ajpheart.1997.273.1.H487

[19] Varga O, Harangi M, Olsson IA, Hansen AK (2010) Contribution of animal models to the understanding of the metabolic syndrome: a systematic overview. Obes Rev 11: 792–807. 10.1111/j.1467-789X.2009.00667.x 19845867

[20] Litten-Brown JC, Corson AM, Clarke L (2010) Porcine models for the metabolic syndrome, digestive and bone disorders: a general overview. Animal 4: 899–920. 10.1017/S1751731110000200 22444262

[21] Roberts RM, Smith GW, Bazer FW, Cibelli J, Seidel GE Jr., Bauman DE, et al (2009) Research priorities. Farm animal research in crisis. Science 324: 468–469. 10.1126/science.1168521 19390030

[22] van der Laan JW, Brightwell J, McAnulty P, Ratky J, Stark C, Steering Group of the RP (2010) Regulatory acceptability of the minipig in the development of pharmaceuticals, chemicals and other products. J Pharmacol Toxicol Methods 62: 184–195. 10.1016/j.vascn.2010.05.005 20601024

