# Supplementary material for: Liver Injury and Fibrosis Induced by Dietary Challenge in the Ossabaw Miniature Swine
Source: PLoS One. 2015 May 15;10(5):e0124173. doi: 10.1371/journal.pone.0124173 (PMC4433111; doi:10.1371/journal.pone.0124173)
Supplement: S1 Table — Morphometric characteristics of swine in the control group (n = 6) and NASH diet group (n = 6) at baseline and at weeks 8, 16 and 24. Mean body circumference (a measurement analogous to waist circumference) was significantly increased in the NASH diet group compared to the control group at weeks 8 and 16 (p = 0.002 and 0.012, respectively). Shown is the mean ± standard deviation. (DOCX) [file pone.0124173.s001.docx]

**Supplemental Table 1: Morphometric characteristics of swine in the control group (n=6) and NASH diet group (n=6) at baseline and at weeks 8, 16 and 24**

|  | **Baseline** | | | **Week 8** | | | **Week 16** | | | **Week 24** | | |
| --- | --- | --- | --- | --- | --- | --- | --- | --- | --- | --- | --- | --- |
|  | **control** | **NASH** | **P** | **control** | **NASH** | **P** | **control** | **NASH** | **^§^P** | **control** | **NASH** | **P** |
| **Body circumference (cm)** | 88.4 ± 5.9 | 89.1 ± 4.5 | 0.793 | 91.3 ± 3.4 | 104.8 ± 5.7 | 0.002 | 95.6 ± 2.8 | 113.8 ± 11.4 | 0.012 | 101.0 ± 3.7 | 116.9 ± 14.8 | 0.087 |
| **Weight (kg)** | 45.4 ± 5.8 | 46.8 ± 5.4 | 0.695 | 52.5 ± 5.7 | 64.2 ± 7.2 | 0.039 | 58.1 ± 5.3 | 82.5 ± 15.6 | 0.020 | 64.6 ± 5.7 | 93.2 ± 20.5 | 0.026 |

Supplemental Table 1: Morphometric characteristics of swine in the control group (n=6) and NASH diet group (n=6) at baseline and at weeks 8, 16 and 24. Mean body circumference (a measurement analogous to waist circumference) was significantly increased in the NASH diet group compared to the control group at weeks 8 and 16 (p=0.002 and 0.012, respectively). Shown is the mean ± standard deviation.
